# Development of a machine learning-derived dendritic cell signature for prognostic stratification in lung adenocarcinoma

**DOI:** 10.3389/fimmu.2025.1621370

**Published:** 2025-06-09

**Authors:** Fang Zhou, Meng Wang, Zheng Wang, Wei Li, Xike Lu

**Affiliations:** ^1^ Tianjin Chest Hospital, Tianjin University, Tianjin, China; ^2^ Clinical School of Thoracic, Tianjin Medical University, Tianjin, China

**Keywords:** LUAD, scRNA-seq, DCs, TME, machine learning

## Abstract

**Background:**

Lung adenocarcinoma (LUAD), the most common histological subtype of lung cancer, demonstrates significant intertumoral heterogeneity. While dendritic cells (DCs) are essential mediators of antitumor immunity, their transcriptional diversity and prognostic value in LUAD remain underexplored.

**Methods:**

We constructed a cellular atlas by integrating single-cell RNA sequencing (scRNA-seq) data from LUAD and normal tissues, emphasizing dendritic cells. High-dimensional weighted gene co-expression network analysis (hdWGCNA) and pseudotime analysis were utilized to identify functional modules and lineage trajectories. A dendritic cell-related signature (DCRS) was constructed using multiple machine learning algorithms (Lasso-Cox, RSF, CoxBoost, Stepwise-Cox), and its prognostic performance was validated in seven external cohorts. Immune landscape, genomic instability, drug sensitivity, and immunotherapy response were further analyzed. The functional role of PLEK2, a DCRS hub gene, was validated in clinical samples and LUAD cell lines.

**Results:**

We identified six DC clusters with distinct developmental states and transcriptional programs. The M2 module was enriched in prognostically relevant clusters and used to derive the DCRS. Patients in the high-DCRS group exhibited worse prognosis, lower immune infiltration, higher chromosomal instability and tumor mutation burden, and reduced responsiveness to immunotherapy. Drug sensitivity analysis revealed that the low-DCRS group was more responsive to multiple chemotherapeutic agents. Functional validation confirmed that *PLEK2* was overexpressed in LUAD tissues and promoted tumor cell proliferation, migration, and colony formation.

**Conclusion:**

We established a novel DCRS with robust prognostic and predictive value in LUAD. This work highlights the pivotal role of dendritic cell programs in shaping the tumor microenvironment and provides potential targets for improving precision immunotherapy.

## 1 Introduction

LUAD is the most prevalent subtype of non-small cell lung cancer (NSCLC) and a major contributor to cancer-related mortality worldwide (1, 2). Despite remarkable progress in targeted therapies and immune checkpoint inhibitors (ICIs), the prognosis of LUAD patients remains unsatisfactory, largely due to substantial intratumoral heterogeneity and complex immune evasion mechanisms (3).

The tumor immune microenvironment (TME) plays a decisive role in tumor progression, therapeutic resistance, and patient survival (4). Among immune cell populations, DCs act as professional antigen-presenting cells that initiate and regulate adaptive immune responses (5). DCs are essential for priming naïve T cells, promoting cytotoxic lymphocyte activation, and orchestrating immunological memory (6, 7). In the TME, however, DC function can be profoundly altered, leading to impaired antigen presentation, T cell dysfunction, and immune escape (8, 9). Emerging evidence indicates that distinct DC subsets may either foster effective antitumor immunity or, conversely, contribute to an immunosuppressive milieu, depending on their maturation state and transcriptional programming (10). Nevertheless, the precise heterogeneity, functional dynamics, and prognostic relevance of DCs in LUAD remain incompletely understood.

scRNA-seq has revolutionized our ability to dissect complex cellular ecosystems within tumors. Unlike bulk RNA sequencing, scRNA-seq captures the transcriptomic profiles of individual cells, enabling high-resolution characterization of rare immune subsets, developmental trajectories, and functional states (11, 12). By integrating scRNA-seq with computational approaches such as pseudotime modeling and gene co-expression network analysis, it becomes possible to systematically map the landscape of DCs and identify clinically relevant transcriptional programs (13).

In this study, we leveraged integrated scRNA-seq datasets from LUAD tumors and normal lung tissues to focus on the dendritic cell compartment. Through trajectory inference and hdWGCNA, we identified functional modules associated with DC states. Based on these findings, we constructed a DCRS and validated its prognostic utility across multiple independent cohorts. Furthermore, we explored the relationship between DCRS and immune infiltration, genomic instability, therapeutic response, and functionally validated the role of the hub gene *PLEK2*. Our work provides new insights into DC-mediated immune regulation in LUAD and proposes DCRS as a promising biomarker for clinical stratification.

## 2 Methods

### 2.1 Data acquisition

Two single-cell RNA sequencing datasets were utilized in this study. The first dataset, GSE171145 (https://www.ncbi.nlm.nih.gov/geo/query/acc.cgi?acc=GSE171145), includes 9 LUAD samples with EGFR mutations (14). The second dataset was obtained from CodeOcean (https://codeocean.com/capsule/8321305/tree/v1), comprising 10 LUAD samples and 10 normal lung tissue samples. Transcriptomic data for model training and validation were sourced from The Cancer Genome Atlas (TCGA, https://portal.gdc.cancer.gov/repository) and the Gene Expression Omnibus (GEO, https://www.ncbi.nlm.nih.gov/geo/), with detailed information provided in Appendix 1. Immune therapy efficacy data were obtained from the following platforms: The Cancer Immunome Atlas (TCIA, https://tcia.at/patients), Tumor Immune Dysfunction and Exclusion (TIDE, https://tide.dfci.harvard.edu). These datasets were used to assess the response to immunotherapy.

### 2.2 Single-cell data preprocessing and cell annotation

In this study, single-cell RNA sequencing data were processed and analyzed using the Seurat(v 4.4.0) package (15). The dataset consisted of two batches: the first batch was in custom format, and the second batch was in CellRanger 10X format. The custom format data were loaded by reading the cellname.list.txt.gz and counts.tsv.gz files to construct Seurat objects, while the CellRanger 10X data were read directly from the corresponding format to create Seurat objects. After preprocessing, all Seurat objects were merged. During the quality control stage, in addition to removing the effects of mitochondrial genes, the percentage of hemoglobin genes was also filtered to ensure data quality. Data normalization was performed using NormalizeData, followed by the selection of 3000 variable genes for downstream analysis. To correct for potential batch effects arising from differences in data source and preprocessing formats, we applied the RunHarmony function from the Harmony package. And cell cycle scoring was conducted with the CellCycleScoring function. Dimensionality reduction was performed using principal component analysis (PCA) and UMAP to visualize the distribution and structure of cell populations. Cell annotation was carried out by combining the GPTCellType (v 1.0.1) package (16) and manual labeling.

### 2.3 Cell-cell communication analysis and network construction

To investigate the interactions between cells, cell-cell communication analysis was performed using the CellChat (v 1.6.1) package (17). First, a CellChat object was constructed based on single-cell RNA sequencing data, and the label information for each cell population was added to the object. By selecting ligand-receptor pathways from the CellChatDB.human database, overexpressed ligands and receptors, as well as their interactions, were identified. The inference of cell communication was achieved by calculating the communication probability for each ligand-receptor pair, followed by projecting the data onto a protein-protein interaction (PPI) network. To further explore the biological significance of cell-cell communication, communication probabilities at the signaling pathway level were calculated, and network data were integrated to generate communication networks between cell populations. Network centrality analysis was performed to identify the signaling pathways playing a key role in the communication network.

### 2.4 Dendritic cell pseudotime analysis and prognostic analysis

Dendritic cells were extracted from the overall Seurat object using the subset function, followed by dimensionality reduction, clustering, and other analytical processes. Pseudotime analysis was primarily conducted using the SCP package (v 0.5.6). In this process, the selected dendritic cell populations were assigned to different lineages, and their developmental trajectories were inferred using the RunSlingshot function. The results of pseudotime analysis were visualized using FeatureDimPlot and DynamicHeatmap, revealing gene expression changes of dendritic cells at different developmental stages. Additionally, dendritic cell marker genes were identified using the FindAllMarkers function.

Subsequently, in the TCGA-LUAD dataset, the ssGSEA algorithm (18) was applied to calculate a score for each patient, estimating the enrichment level of different dendritic cell populations in individual patients. Based on these scores, Kaplan-Meier survival analysis was performed using the survminer and survival packages to assess the prognostic impact of various dendritic cell populations on patient survival. Finally, pathway enrichment analysis was conducted using the GSEA algorithm (19) to explore the differences in pathway activity between dendritic cell subpopulations.

### 2.5 hdWGCNA analysis

The hdWGCNA (v 0.4.05) (20, 21) analysis was performed by preprocessing the Seurat object using the SetupForWGCNA function and selecting appropriate genes for co-expression network analysis. The k-Nearest Neighbors (KNN) algorithm was employed to aggregate similar cells into metacells, and their average gene expression levels were calculated. Subsequently, a co-expression network was constructed by selecting an optimal soft threshold, and modules were identified using the blockwiseConsensusModules function. The gene expression features of each module were characterized by module eigengenes (MEs). The top 25 hub genes for each module were further computed, and their expression levels were assessed using the UCell method. The analysis results were visualized using UMAP, heatmaps, and violin plots to illustrate the gene expression patterns and functional characteristics of dendritic cell populations.

### 2.6 Construction of dendritic cell-related signature

Differential gene analysis was first performed on the TCGA-LUAD dataset. The limma(v 3.60.4) package (22, 23) was used to compare gene expression between tumor and normal samples. Significant genes were selected based on the criteria of a P-value less than 0.05 and an absolute logFC greater than 0.8. These differentially expressed genes were then intersected with dendritic cell-related marker genes and the module genes identified through hdWGCNA analysis to form the final candidate gene set. Subsequently, univariate Cox regression analysis was employed to identify prognosis-related variables. Variables that were statistically significant in the univariate analysis were selected as candidate features for the subsequent construction of the prognostic model. For the model construction, multiple machine learning algorithms were applied, including LASSO regression (24), CoxBoost regression (25), and Random Forest (26). The performance of these models was evaluated using cross-validation and C-index, with the best-performing algorithm chosen as the final model. The training and validation sets were stratified into risk groups based on the median risk score derived from the model. Kaplan-Meier survival curves (K-M curves) and ROC curves were utilized to assess the predictive efficacy of the model. Furthermore, the model’s reliability was validated by comparing it with 114 previously published LUAD prognostic models.

### 2.7 Comprehensive evaluation of key pathways in LUAD

In this study, enrichment analyses were performed using GSVA (v 1.52.3) (27), GSEA (v 1.66.0), and ssGSEA methods. First, gene sets from the Hallmark pathways were extracted using the msigdbr package, and GSVA was applied to calculate enrichment scores for the TCGA-LUAD dataset samples. Specifically, GSVA computed enrichment scores for each sample across different gene sets, reflecting the activity of samples in various pathways. Next, differential analysis of GSVA scores between high-risk and low-risk groups was conducted using the limma package, identifying significantly enriched pathways. Subsequently, GSEA analysis was performed on the differentially expressed genes in the TCGA-LUAD dataset. The logFC values of genes were calculated by comparing the gene expression differences between high-risk and low-risk groups. GSEA based on KEGG pathways was then carried out to identify pathways associated with LUAD prognosis. In the ssGSEA analysis, immune-related gene sets were selected to assess the activity of specific immune pathways, and the pathway enrichment scores for each sample were computed. The ssGSEA method provided a personalized pathway score for each sample, evaluating the relationship between immune pathways and the prognosis of LUAD patients.

### 2.8 Immune landscape and prognostic implications in LUAD based on risk group stratification

The results from seven immune cell infiltration algorithms (including CIBERSORT (28), MCPCOUNTER (29), XCELL (30), etc.) were first downloaded from the TIMER2.0 database (31). These algorithms were used to assess the immune cell abundance in each sample. Based on the risk stratification of the patients (high-risk and low-risk groups), the ComplexHeatmap(v 2.20.0) package (32) was employed to visualize the differences in immune cell infiltration between the risk groups. Subsequently, the Estimate algorithm was used to evaluate the immune and stromal scores of the samples. This algorithm analyzes gene expression data from each sample to calculate the immune score and tumor score, providing quantitative information on the immune and stromal components of the tumor microenvironment, further exploring the differences in immune microenvironment between the high-risk and low-risk groups. Next, the ssGSEA algorithm was used to assess the differences in immune-related function between the high-risk and low-risk groups. By calculating the enrichment scores of immune-related pathways in each sample, ssGSEA evaluated the activation levels of various pathways, revealing the differences in immune responses between the risk groups.

### 2.9 qRT-PCR analysis

Quantitative real-time PCR (qRT-PCR) was performed to validate the expression levels of key model genes. Total RNA was extracted using TRIzol reagent and reverse-transcribed into cDNA using a commercial synthesis kit. qRT-PCR assays were conducted on an ABI QuantStudio system with three technical replicates per sample. GAPDH was used as the internal control, and gene expression levels were calculated using the ΔCt method (ΔCt = Ct_target − Ct_GAPDH). Relative expression levels were expressed as 2^-ΔCt. The primer sequences for the target gene *PLEK2* were as follows:Forward primer: CCGAAGCATGGGAGCCATT; Reverse primer: AGTGCTCAGGCTAATTTCTTCC.

### 2.10 Cell culture and siRNA transfection

The human lung adenocarcinoma cell lines A549 and H1299 were obtained from an authenticated cell bank and cultured in RPMI-1640 medium supplemented with 10% fetal bovine serum (FBS) and 1% penicillin-streptomycin at 37°C in a humidified incubator containing 5% CO_2_. Cells in the logarithmic growth phase were subjected to transfection. Small interfering RNAs (siRNAs) targeting the gene of interest were transfected using Lipofectamine RNAiMAX reagent (Invitrogen) following the manufacturer’s protocol. The sequences of siRNAs targeting *PLEK2* were as follows:

si*PLEK2*_1:5′-ACCUCUUCAAAGUGAUUACUA-3′;si*PLEK2*_2:5′-CCAGCUUUCCUGCAUUACUAU-3′. Cells were harvested 48 hours post-transfection for subsequent analyses.

### 2.11 Transwell migration and invasion assays

Cell migration and invasion abilities were assessed using Transwell chambers (8 μm pore size, Corning). For migration assays, cells were seeded in serum-free medium into the upper chambers; for invasion assays, Matrigel-coated chambers were used. After 24 hours of incubation, cells on the lower membrane surface were fixed with methanol, stained with crystal violet, and counted in five random fields. All experiments were performed in triplicate.

### 2.12 CCK-8 cell proliferation assay

Cells were seeded into 96-well plates at appropriate densities and cultured continuously for 7 days. CCK-8 reagent (Dojindo) was added every 24 hours, and absorbance at 450 nm was measured after 1–2 hours of incubation. Each group was assayed in triplicate, and experiments were independently repeated three times to generate proliferation curves.

### 2.13 Colony formation assay

Cells were seeded into 6-well plates at a low density (500–1000 cells per well) and cultured for approximately 10–14 days until visible colonies formed. Colonies were then gently washed with PBS, fixed with 4% paraformaldehyde for 15 minutes, and stained with 0.1% crystal violet for 20 minutes. Excess dye was washed off, and colonies were photographed and counted.

### 2.14 Statistical analysis

All statistical analyses were performed using R software (version 4.2.1) and GraphPad Prism (version 9.0). Continuous variables were expressed as mean ± standard deviation (SD). Comparisons between two groups were conducted using Student’s t-test or nonparametric tests, as appropriate. Survival analyses were performed using the Kaplan–Meier method, and differences between groups were evaluated using the log-rank test. Correlation analyses were conducted using Pearson or Spearman correlation coefficients depending on data distribution. For multiple testing correction in differential expression and enrichment analyses, the Benjamini–Hochberg false discovery rate (FDR) method was applied unless otherwise specified. All statistical tests were two-sided, and a P-value less than 0.05 was considered statistically significant. Statistical significance was indicated by asterisks, with * for P < 0.05, ** for P < 0.01, and *** for P < 0.001.

## 3 Results

### 3.1 Single-cell clustering and immune microenvironment cell–cell interaction profiling

In this study, rigorous quality control was applied to the integrated single-cell RNA sequencing data. Cells were filtered based on the following criteria: number of detected genes (nFeature) between 500 and 10,000, total UMI counts (nCount) between 1,000 and 100,000, and mitochondrial gene percentage (pMT) below 40%. To minimize confounding effects from cell cycle variability, cell cycle-related gene expression was regressed out using the ScaleData function in Seurat, ensuring more reliable downstream clustering analysis (see **Supplementary Figure 1**).

**Figure 1:**
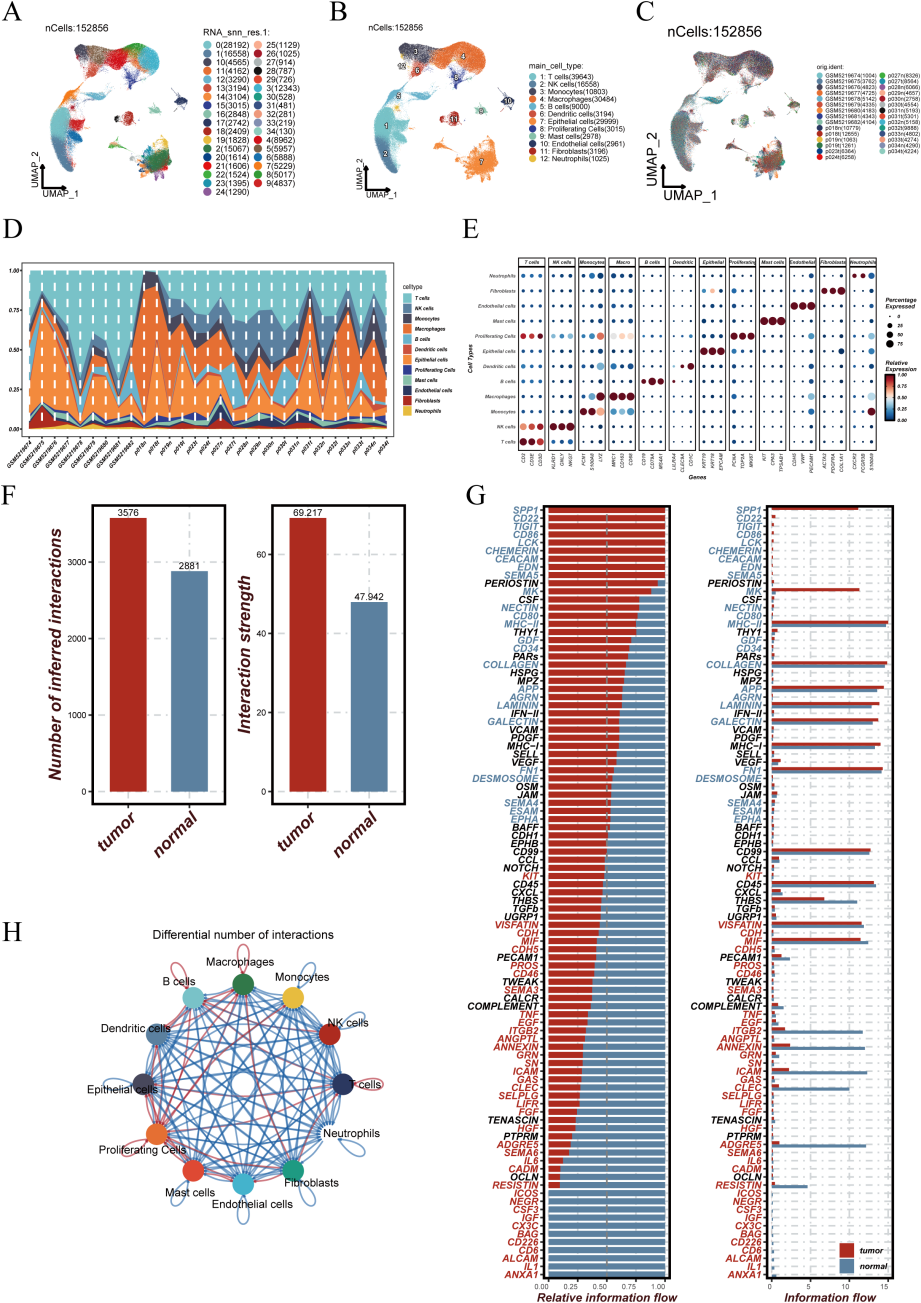
Construction of the single-cell atlas and analysis of cell–cell communication differences between tumor and normal tissues. **(A)** UMAP projection of 152,856 single cells grouped into 35 distinct clusters. **(B)** Cell types were annotated based on canonical marker genes, identifying epithelial, immune, and stromal cell populations. **(C)** Spatial distribution of cells across individual patient samples. **(D)** Proportional abundance of each cell type across samples, illustrating inter-patient heterogeneity in cellular composition. **(E)** Expression levels of representative marker genes across annotated cell types, confirming annotation accuracy and specificity. **(F)** Bar plots showing that the number and strength of inferred intercellular interactions were significantly higher in tumor tissues compared to normal controls. **(G)** Differential analysis of signaling pathways highlights tumor-enriched pathways such as COLLAGEN and PERIOSTIN, with relative information flow indicating altered communication dynamics. **(H)** Network diagram of intercellular communication across major cell types. Node size indicates the number of interactions involving each cell type, while edge thickness represents interaction strength, demonstrating enhanced cross-talk among immune and non-immune populations in the tumor microenvironment.

After quality control, a total of 152,856 high-quality cells were retained. Uniform Manifold Approximation and Projection (UMAP) was performed for dimensionality reduction, resulting in the identification of 35 distinct cell clusters (**Figure 1A**). Based on canonical marker gene expression, cell types were annotated, revealing major populations such as epithelial cells, immune cells, fibroblasts, and others (**Figure 1B**). The spatial distribution of cells across individual patient samples is visualized in **Figure 1C**, demonstrating the diverse cellular compositions among different tissues. The abundance of each cell type per sample is shown in **Figure 1D**, highlighting marked inter-individual heterogeneity in the tumor microenvironment. **Figure 1E** displays representative marker gene expression patterns across annotated cell types, supporting the accuracy and specificity of the cell classification. To further investigate intercellular communication within the tumor microenvironment, CellChat analysis was conducted separately for tumor and normal tissues. As shown in **Figure 1F**, both the number of inferred interactions and the overall interaction strength were markedly higher in tumor samples compared to normal tissues, suggesting more active cellular crosstalk in the tumor context. At the signaling pathway level, differential information flow was observed across various pathways (**Figure 1G**), with several pathways showing increased or decreased signaling activity in tumors. **Figure 1H** illustrates the differential communication networks between cell types, depicting how interactions among epithelial, immune, and stromal cells are reorganized in the tumor microenvironment.

### 3.2 Trajectory inference and functional heterogeneity of dendritic cells

**Figure 2:**
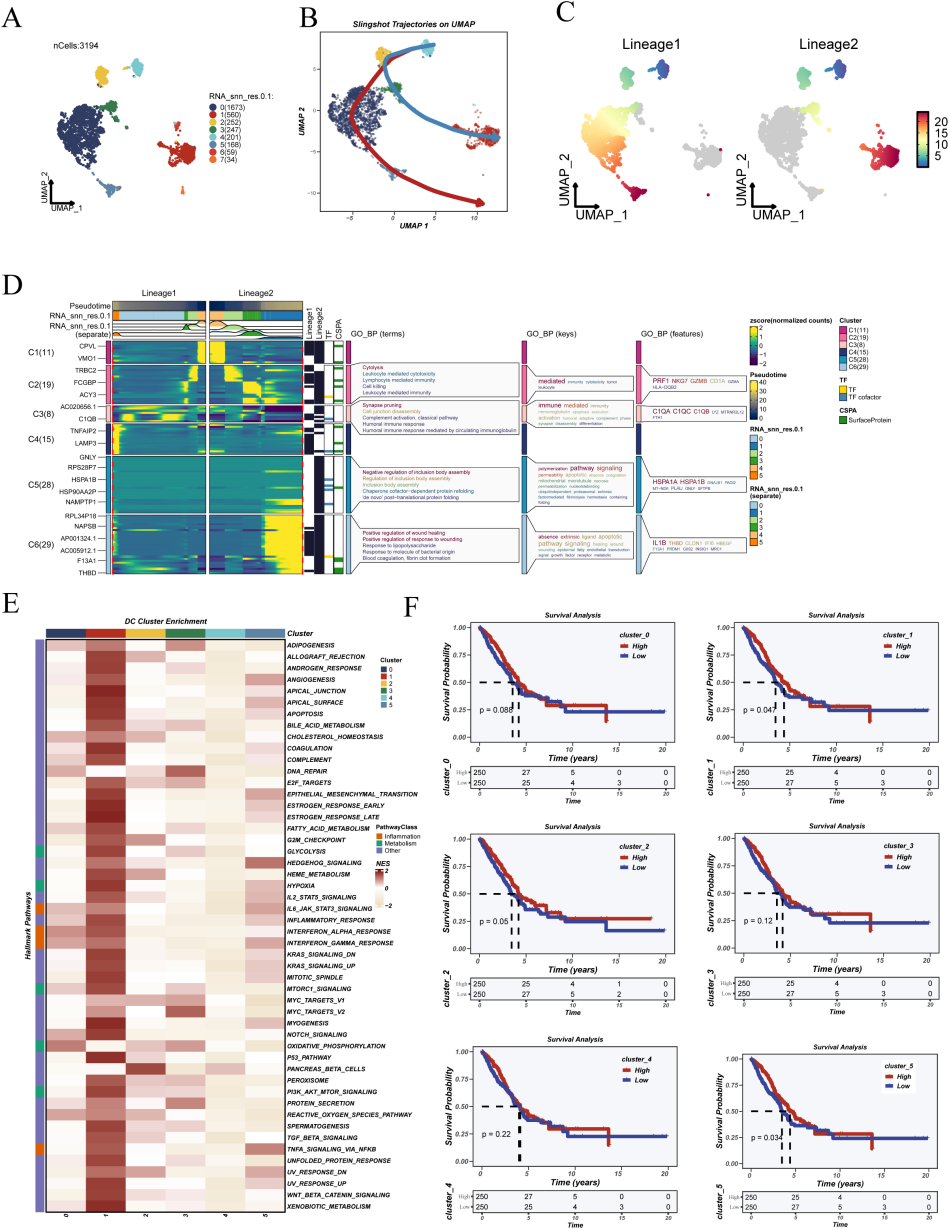
Pseudotime trajectory inference, functional enrichment, and prognostic analysis of dendritic cells. **(A)** UMAP visualization showing the clustering results of dendritic cells. **(B)** Pseudotime trajectories inferred using the SlingShot algorithm, illustrating the differentiation progression of dendritic cells. **(C)** Two distinct differentiation lineages (Lineage 1 and Lineage 2) were identified based on pseudotime analysis. **(D)** Heatmap and GO enrichment of dynamic genes along both lineages, revealing functional transitions during dendritic cell maturation. **(E)** Hallmark pathway enrichment analysis of six dendritic cell clusters, indicating distinct immune and metabolic programs. **(F)** Kaplan–Meier survival curves of LUAD patients in the TCGA cohort, stratified by proportions of dendritic cell subclusters or pseudotime-defined lineages, demonstrating the prognostic relevance of dendritic cell heterogeneity.

To explore the developmental dynamics and functional heterogeneity of dendritic cells, this population was extracted and analyzed independently. UMAP visualization revealed distinct clustering patterns among dendritic cells (**Figure 2A**). Using the SlingShot algorithm, two principal pseudotime trajectories were identified, suggesting bifurcating differentiation paths toward distinct cellular states (**Figures 2B, C**).

Genes associated with these trajectories were subjected to GO enrichment analysis, which highlighted processes such as immune regulation, chemokine signaling, and antigen presentation (**Figure 2D**). Hallmark pathway enrichment (**Figure 2E**) revealed that functional clusters exhibited differential enrichment in key immune and stress-related programs. Specifically, cluster 1 was enriched in immune-activating pathways such as “INTERFERON_ALPHA_RESPONSE” and “TNFA_SIGNALING_VIA_NFKB”, whereas cluster 5 was associated with proliferative signatures including “G2M_CHECKPOINT” and “MYC_TARGETS_V1”. To investigate their clinical relevance, the relative abundance of each cluster was quantified per patient in the TCGA-LUAD cohort and stratified by median values. Kaplan–Meier survival analyses revealed that clusters 1 and 5 were significantly associated with overall survival outcomes (**Figure 2F**), suggesting their potential as prognostic indicators.

### 3.3 Identification of co-expression modules in dendritic cells via hdWGCNA

**Figure 3:**
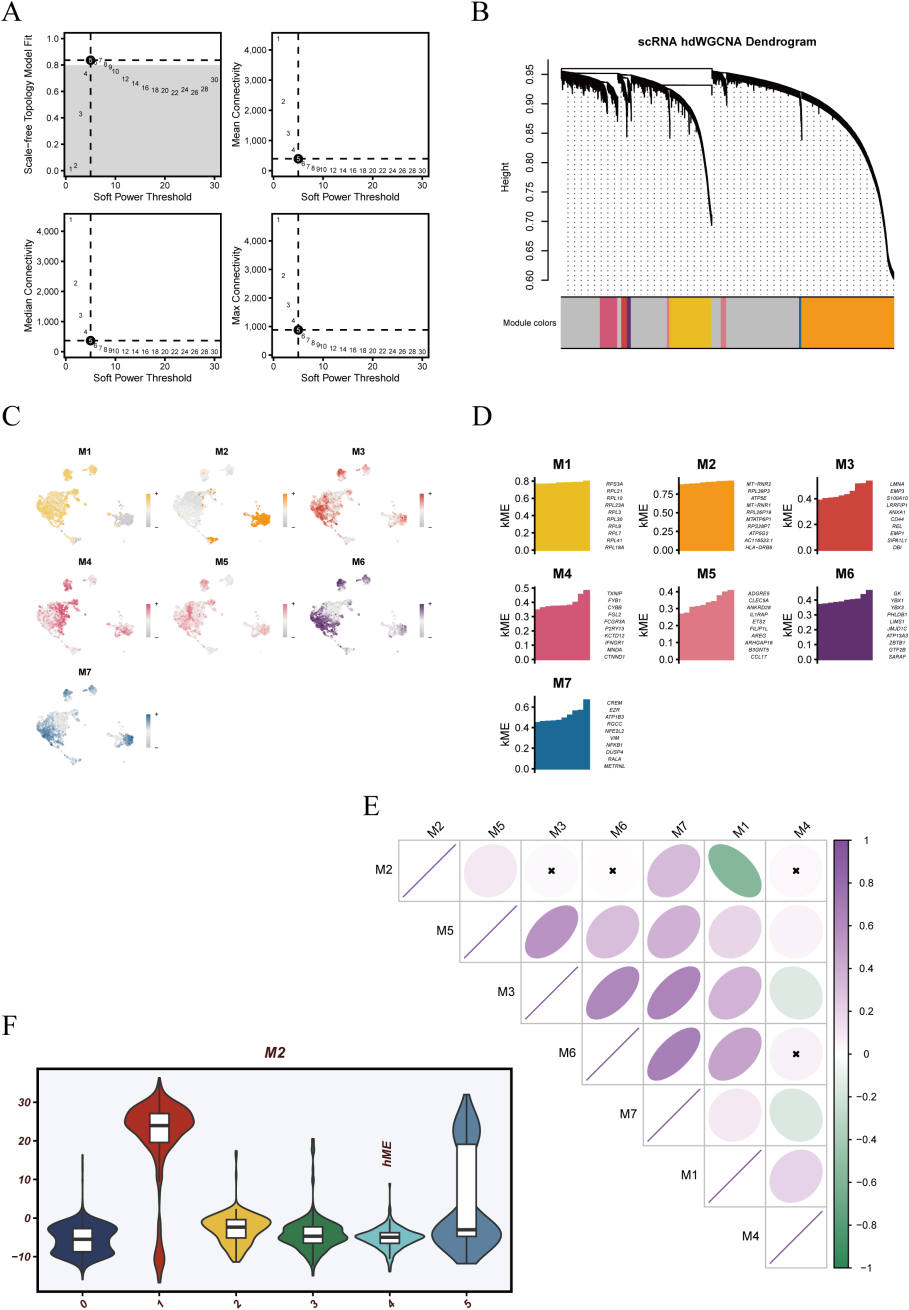
hdWGCNA-based weighted gene co-expression network analysis of dendritic cells. **(A)** Soft-threshold power selection plots used to identify an optimal power value ensuring scale-free topology for network construction. **(B)** Hierarchical clustering dendrogram of genes with module color annotations. Seven co-expression modules (M1–M7) were identified. **(C)** UMAP visualization of module eigengene (ME) scores showing the spatial distribution of each module across dendritic cell clusters. **(D)** Bar plots displaying the eigengene expression profiles (MEs) of individual modules, reflecting their module-specific activity patterns. **(E)** Correlation matrix of MEs across all modules, indicating varying degrees of inter-module relationships. **(F)** Violin plot of ME scores for the M2 module across different dendritic cell subpopulations, suggesting potential functional specificity of this module.

To identify transcriptional programs within dendritic cells, we constructed a gene co-expression network using the hdWGCNA framework. A soft-thresholding power of 5 was selected based on scale-free topology criteria, as determined by network diagnostics (**Figure 3A**). With this threshold, a weighted network was built and hierarchical clustering revealed seven distinct gene modules, each represented by a unique color (**Figure 3B**). Intra-modular gene co-expression networks were further visualized (**Supplementary Figure 2**), revealing that most modules exhibited densely interconnected structures, suggesting strong functional coherence among member genes.

Spatial distribution of module eigengene (ME) scores projected onto UMAP embedding revealed distinct expression patterns across the cellular landscape (**Figure 3C**). M2 exhibited the most pronounced spatial concentration, aligning with Cluster 1 and Cluster 5 regions. M3 and M6 also showed relatively focused expression zones, whereas M1 and M4 were more diffusely distributed, suggesting broader functional activity. The expression levels of module eigengenes (MEs) varied across individual cells, reflecting differential module activity (**Figure 3D**). Correlation analysis between modules showed a strong negative association between M2 and M1 (**Figure 3E**), suggesting potentially antagonistic functional roles. Notably, M2 module activity was significantly elevated in Cluster 1 and Cluster 5 (**Figure 3F**), indicating that this module may underlie specific functional programs in these two dendritic cell subsets and warranting further investigation.

### 3.4 Construction of a dendritic cell-related signature via multi-omics integration and machine learning

**Figure 4:**
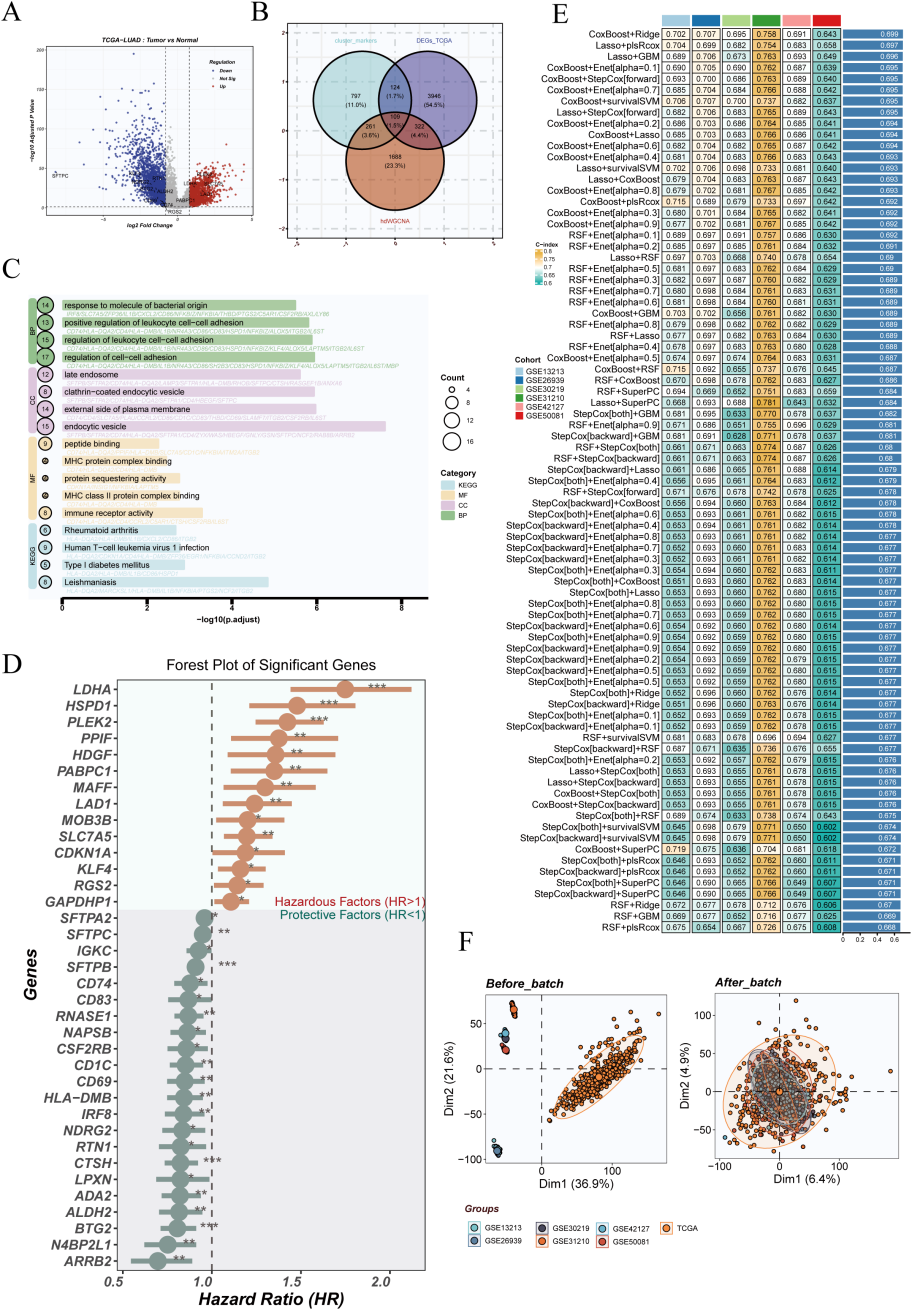
Differential expression analysis and machine learning-based prognostic model construction. **(A)** Volcano plot displaying differentially expressed genes between tumor and normal samples in the TCGA-LUAD dataset. **(B)** Venn diagram showing the intersection of DEGs, marker genes from dendritic cell clusters 1 and 5, and genes from the M2 module identified by hdWGCNA. **(C)** GO and KEGG enrichment analyses of the intersecting genes reveal their involvement in immune responses, cytokine signaling, and metabolic pathways. **(D)** Univariate Cox regression analysis of the intersecting genes, with a forest plot highlighting significant prognostic genes (HR >1 as risk factors; HR <1 as protective factors). **(E)** Prognostic models constructed using various machine learning algorithms, including Lasso-Cox, Random Survival Forest (RSF), CoxBoost, and others. The performance (C-index) was assessed across multiple validation cohorts. **(F)** Principal component analysis (PCA) plots showing sample distribution before and after batch effect correction using the sva algorithm, demonstrating improved integration across TCGA and GEO datasets post-correction.

To develop a DCRS in LUAD, we first performed differential gene expression analysis in the TCGA-LUAD dataset, identifying genes with |log2FC| > 0.8 and FDR < 0.05 (**Figure 4A**). These genes were intersected with markers from Cluster 1 and Cluster 5 and M2 module genes derived from hdWGCNA, yielding a total of 108 overlapping genes (**Figure 4B**).

GO and KEGG enrichment analyses revealed that these intersecting genes were predominantly involved in immune regulatory processes, including antigen processing and presentation, MHC complex assembly, and cytokine signaling pathways (**Figure 4C**). Univariate Cox regression analysis identified multiple genes significantly associated with overall survival, such as LDHA and HSP90AA1 as risk factors, and CD86 and HLA-DMB as protective factors (**Figure 4D**).

To construct the DCRS, we systematically applied a panel of machine learning algorithms—including Lasso-Cox, Random Survival Forest (RSF), CoxBoost, and Stepwise-Cox—either individually or in combination. Model performance was evaluated using repeated cross-validation and C-index in seven external cohorts. Among all strategies tested, the combination of CoxBoost with Ridge regularization demonstrated optimal predictive accuracy and robustness (**Figure 4E**).

To correct for technical variability across datasets, the sva algorithm was employed. Principal component analysis revealed clear batch effects prior to correction, which were largely mitigated after adjustment, indicating effective normalization and improved dataset integration (**Figure 4F**).

### 3.5 Internal features and risk stratification of the DCRS signature

To further characterize the distribution of the DCRS signature across patients, the risk score distribution, survival status, and expression patterns of DCRS component genes were visualized (**Supplementary Figure 3A**). Patients with higher risk scores exhibited a markedly increased incidence of death events, concomitant with elevated expression levels of DCRS genes. Correlation analysis between risk scores and the expression of individual DCRS genes (**Supplementary Figure 3B**) revealed strong positive associations, indicating that these genes substantially contributed to the risk stratification. Moreover, analysis of pathological stage distribution between the risk groups (**Supplementary Figure 3C**) demonstrated that patients in the high-risk group were more likely to present with advanced stages (P = 0.001), suggesting that the DCRS signature was associated not only with poor prognosis but also with disease progression.

### 3.6 Generalization and clinical benchmarking of DCRS across multiple datasets

**Figure 5:**
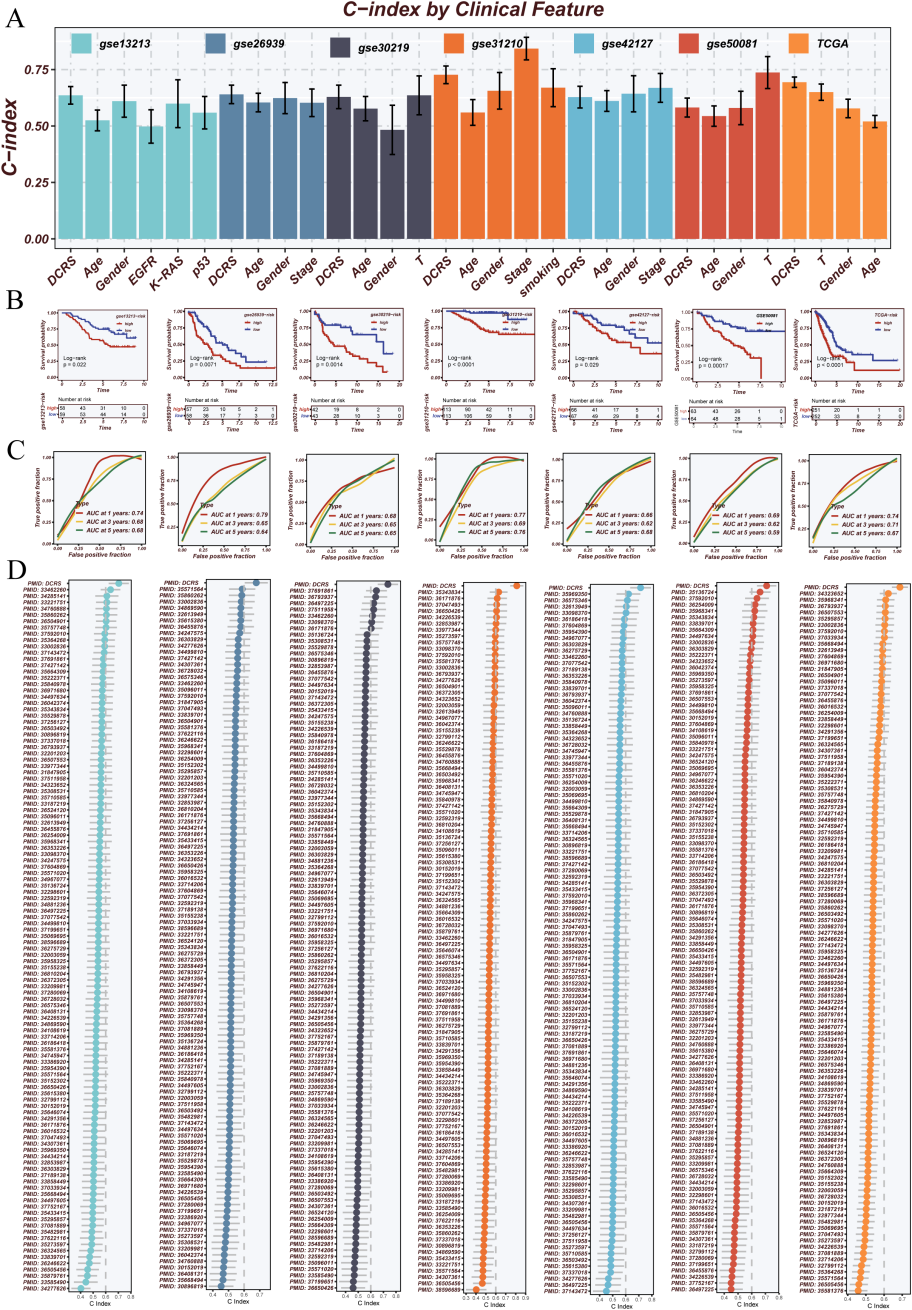
Performance evaluation and benchmarking of the prognostic model. **(A)** C-index comparison of the proposed model with common clinical variables (e.g., Age, Gender, Stage, EGFR status) across multiple external validation datasets. The model consistently outperformed clinical features in prognostic prediction. **(B)** Kaplan–Meier survival analyses demonstrating the model’s ability to stratify patients into high- and low-risk groups with significantly different survival outcomes in various cohorts. **(C)** Time-dependent ROC curves evaluating the model’s predictive performance for 1-, 3-, and 5-year overall survival (OS). The area under the curve (AUC) values indicate robust prognostic accuracy. **(D)** Systematic comparison of the proposed model against 114 previously published mRNA- and lncRNA-based prognostic signatures using C-index across seven datasets. The model exhibited superior or comparable performance, highlighting its generalizability and robustness.

To comprehensively evaluate the prognostic performance of DCRS, we compared its C-index with conventional clinical features—including age, gender, stage, and EGFR mutation status—across seven independent validation cohorts. As shown in **Figure 5A**, DCRS consistently outperformed these clinical variables in most datasets, demonstrating superior prognostic capability and robustness.

Kaplan–Meier survival analyses across multiple cohorts confirmed that DCRS effectively stratified patients into high- and low-risk groups with significantly different survival outcomes (**Figure 5B**). Time-dependent ROC analyses further validated the model’s predictive accuracy at 1-, 3-, and 5-year survival intervals, with DCRS maintaining high AUC values across all timepoints (**Figure 5C**).

Moreover, a systematic comparison was conducted between DCRS and 114 previously published prognostic signatures based on mRNA and lncRNA features. DCRS achieved higher C-index values across various datasets, underscoring its broad generalizability and potential for clinical application (**Figure 5D**).

### 3.7 Functional and immune pathway enrichment analysis based on DCRS stratification

**Figure 6:**
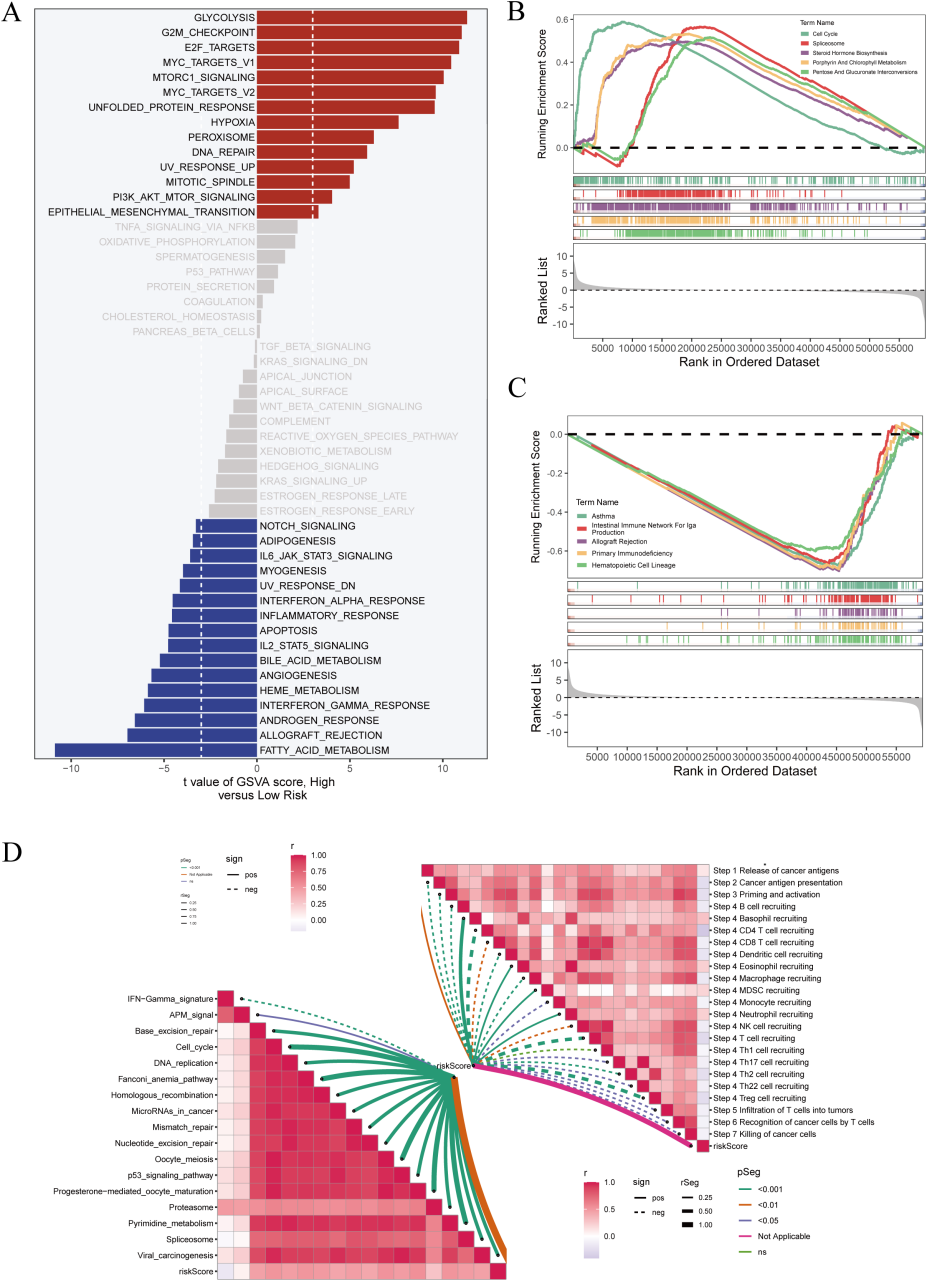
Pathway enrichment analyses of high- and low-risk groups. **(A)** GSVA (Gene Set Variation Analysis) was performed to assess pathway activity across samples. The t-values represent differences in pathway scores between high- and low-risk groups. **(B)** GSEA (Gene Set Enrichment Analysis) was conducted specifically in high-risk patients. Enrichment plots for representative pathways are shown. **(C)** GSEA was similarly applied to low-risk patients, with corresponding enrichment curves displayed. **(D)** ssGSEA (single-sample GSEA) was used to calculate immune pathway activity scores per sample. The left panel shows a network plot of immune-related signatures, while the right panel presents a correlation heatmap among these immune pathways.

To further explore the functional implications of DCRS, gene set variation analysis (GSVA) was conducted between high- and low-risk groups. As shown in **Figure 6A**, the high-risk group was significantly enriched in proliferative and metabolic pathways, including glycolysis, G2M checkpoint, MYC targets, and PI3K/AKT/mTOR signaling. In contrast, the low-risk group showed marked enrichment in immune-related pathways, such as interferon responses, inflammatory signaling, and antiviral immunity.

To validate these findings, GSEA was performed separately in high-risk (**Figure 6B**) and low-risk (**Figure 6C**) subgroups. The results confirmed the distinct enrichment directions of key immune and proliferation-related pathways in the two DCRS-defined populations.

Furthermore, ssGSEA was used to calculate immune-related functional scores for each sample. A correlation network of immune pathways was constructed (**Figure 6D**), revealing coordinated activation of multiple immune processes. Notably, the low-risk group exhibited higher activation of antigen presentation, T-cell stimulation, and interferon-related pathways, supporting a more immunologically active phenotype in this subgroup.

### 3.8 Immunological heterogeneity of the tumor microenvironment across DCRS subgroups

**Figure 7:**
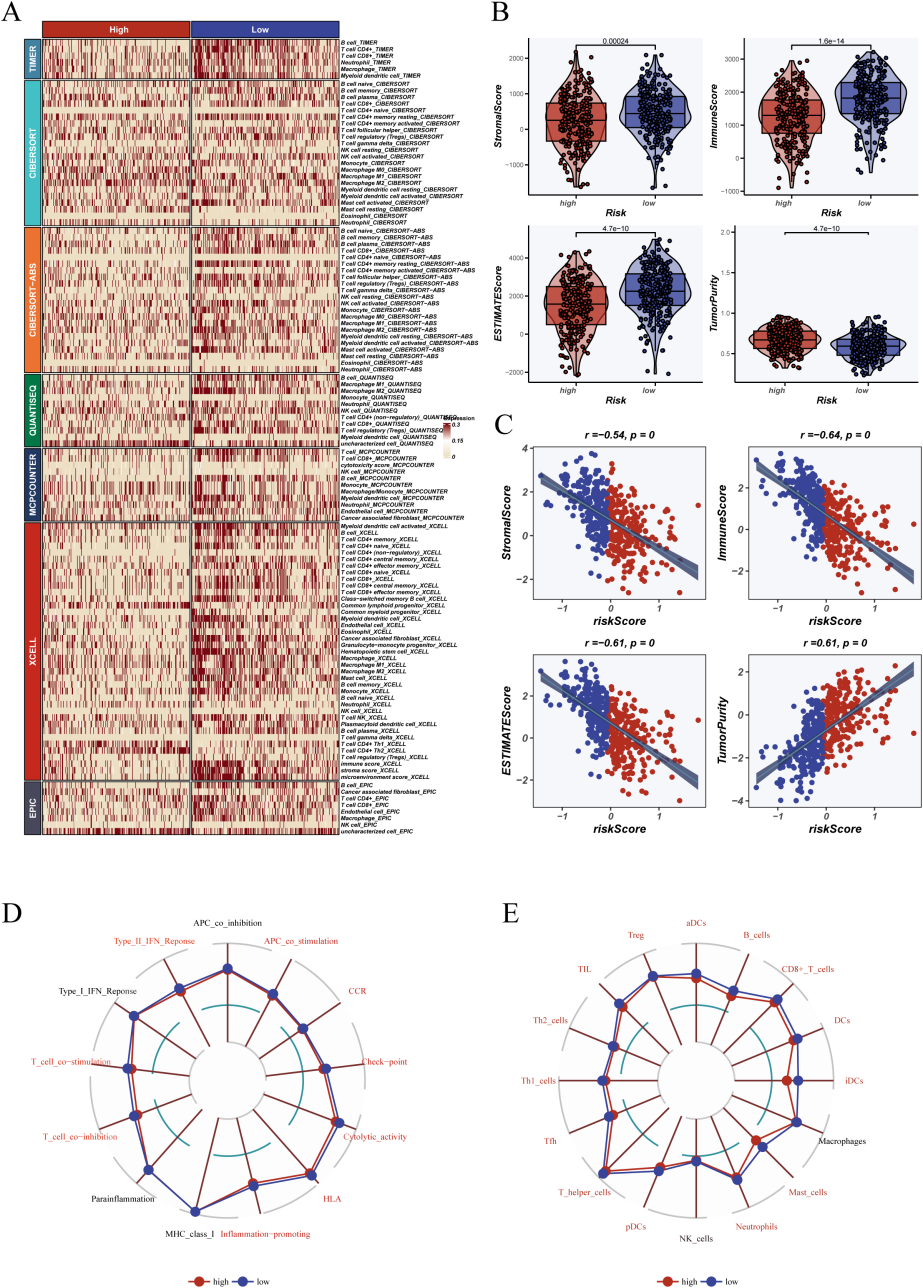
Tumor microenvironment and immune landscape analysis. **(A)** Heatmap illustrating immune cell infiltration scores between high- and low-risk groups across multiple algorithms and databases, including TIMER, CIBERSORT, XCELL, EPIC, MCPcounter, and ssGSEA. **(B)** ESTIMATE algorithm was used to evaluate TME-related indices of tumor samples, including stromal score, immune score, ESTIMATE score, and tumor purity, with comparisons made between risk groups; **(C)** Correlation plots showing the relationship between risk score and each tumor microenvironment index. **(D)** Radar chart depicting immune-related functional differences between groups based on ssGSEA, including antigen-presenting cell activity, type I/II IFN response, and immune co-stimulatory pathways. **(E)** Immune cell infiltration levels for various immune cell subsets (e.g., CD8+ T cells, B cells, macrophages, dendritic cells) between high- and low-risk groups. Variables shown in red font indicate statistical significance (P < 0.05).

To investigate the association between the dendritic cell-related signature (DCRS) and the tumor immune microenvironment, we analyzed immune cell abundance using multiple infiltration estimation platforms (e.g., TIMER, CIBERSORT, XCELL). As shown in **Figure 7A**, the low-risk group exhibited higher levels of infiltration across key immune cell types, including T cells, macrophages, and dendritic cells.

To further investigate functional differences, we performed pathway enrichment analysis using the GSEA algorithm based on KEGG pathway gene sets. As shown in **Figure 6B**, the high-DCRS group exhibited significant enrichment in proliferation- and metabolism-related pathways, such as glycolysis, G2M checkpoint, MYC targets, and PI3K/AKT/mTOR signaling. Conversely, the low-DCRS group showed increased enrichment in immune-related pathways including antigen processing and presentation, interferon-α and -γ responses, and T cell receptor signaling (**Figure 6C**). These findings underscore the functional divergence between the two risk groups and suggest that the low-DCRS subgroup possesses a more immunologically active tumor phenotype.

Functionally, ssGSEA revealed that samples in the low-risk group displayed higher activation of immune processes such as antigen presentation, Type I interferon response, and T cell co-stimulation (**Figure 7D**). Additionally, the low-risk group showed greater infiltration by CD8+ T cells, follicular helper T cells, and dendritic cells (**Figure 7E**), consistent with an immunologically active tumor phenotype.

### 3.9 Association of DCRS with genomic instability features (CNA and TMB)

**Figure 8:**
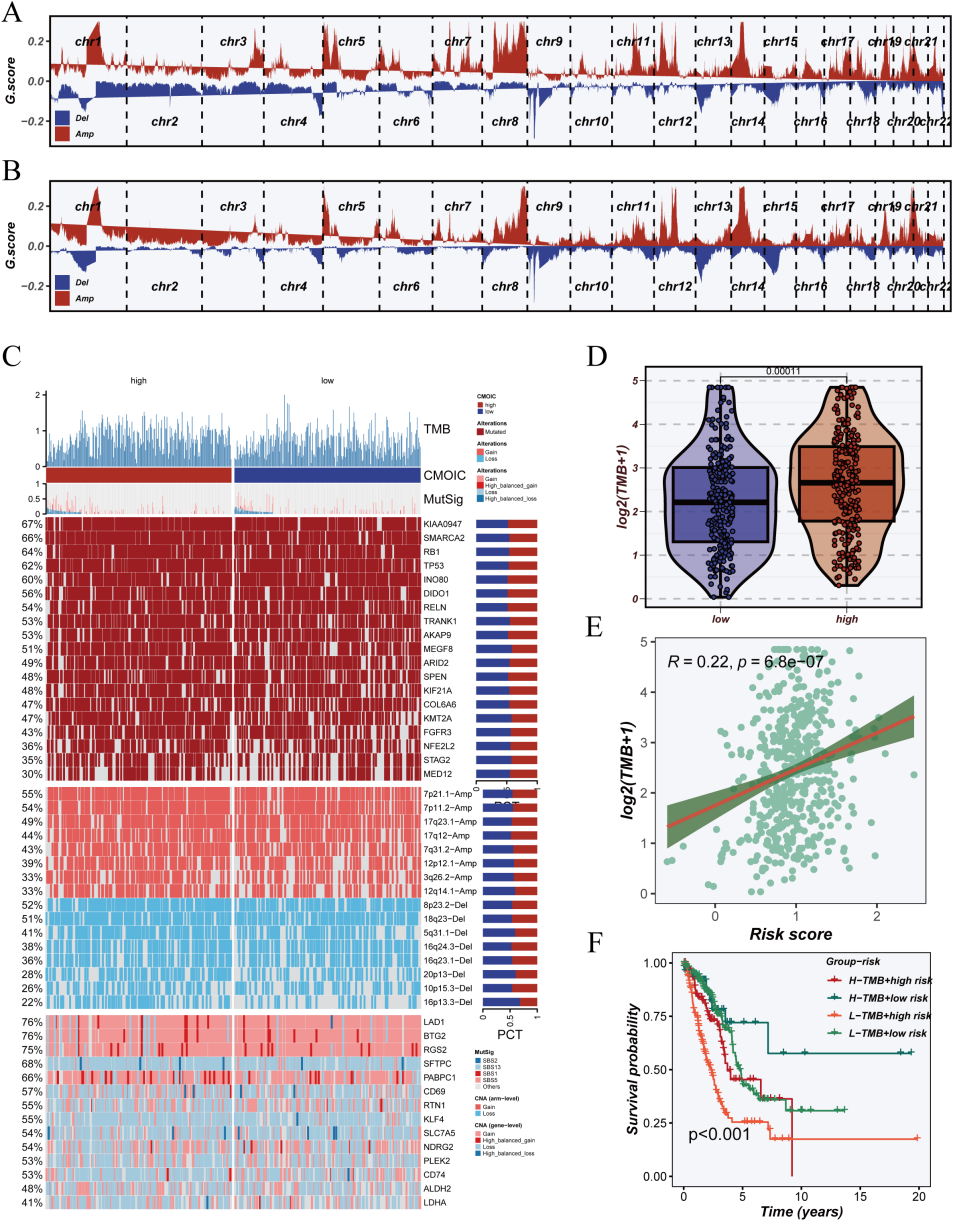
Somatic mutation profiles and association with tumor mutation burden (TMB). **(A)** Genome-wide copy number variation (CNV) landscape in the high-risk group, showing amplifications (Amp) and deletions (Del) across chromosomes. **(B)** CNV landscape in the low-risk group for comparative visualization against the high-risk group. **(C)** Overview of somatic mutation landscape, including TMB scores, significantly mutated genes (MutSig), and frequently mutated genes across high- and low-risk samples. **(D)** Violin plot comparing log2-transformed TMB values between high- and low-risk groups. **(E)** Correlation analysis between risk score and TMB, presented as a scatter plot with linear regression. **(F)** Kaplan–Meier survival analysis based on combined stratification of patients by TMB level and risk score, revealing prognostic differences across four subgroups.

To evaluate the association between the DCRS and genomic instability, we analyzed copy number alterations (CNAs) across the genome in both high- and low-DCRS groups. As illustrated in **Figures 8A, B**, the high-DCRS group showed extensive chromosomal amplifications and deletions, whereas the low-DCRS group exhibited relatively stable genomic profiles, suggesting that elevated DCRS is linked to increased chromosomal instability.

We next compared tumor mutation burden (TMB) between groups. The high-DCRS group displayed significantly higher TMB levels than the low-DCRS group (**Figure 8D**), and correlation analysis confirmed a positive relationship between DCRS riskScore and TMB (R = 0.22, p < 0.001) (**Figure 8E**), indicating that DCRS may reflect mutation accumulation. **Figure 8C** presents the mutation landscape sorted by DCRS classification. Driver genes such as TP53, KRAS, KEAP1, and STK11 were more frequently mutated in the high-DCRS group, and these cases were also enriched for chromosomal gains and losses. Mutation type analysis revealed that missense and nonsense mutations were predominant in the high-DCRS group, highlighting its association with elevated genomic instability.

Joint stratification based on TMB and DCRS revealed a synergistic effect on survival outcomes (**Figure 8F**). Patients in the TMB-low + DCRS-low group exhibited the most favorable prognosis, whereas those in the TMB-high + DCRS-high group had the poorest outcomes. These findings suggest that DCRS provides additional prognostic resolution beyond TMB alone and may assist in refining risk stratification in clinical practice.

### 3.10 Predictive value of DCRS for immune escape and therapeutic responsiveness

**Figure 9:**
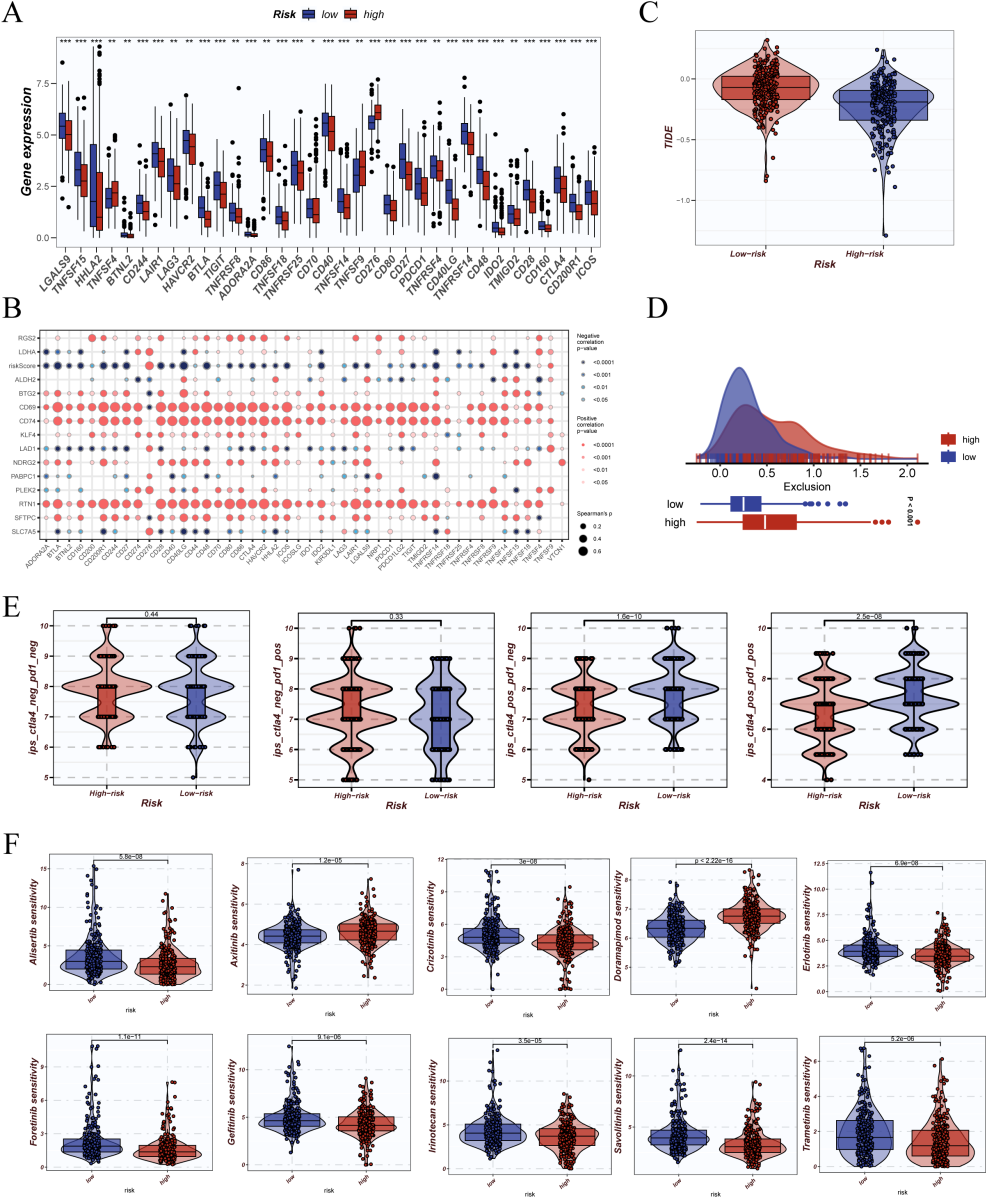
Prediction of immunotherapy response and drug sensitivity analysis. **(A)** Differential expression analysis of immune checkpoint-related genes (e.g., PDCD1, CTLA4, LAG3) between high- and low-risk groups shown as boxplots. **(B)** Correlation matrix of immune checkpoint genes presented as a bubble plot, indicating Pearson correlation coefficients and statistical significance. **(C)** Tumor Immune Dysfunction and Exclusion (TIDE) scores compared between risk groups to assess predicted immunotherapy response. **(D)** Distribution of TIDE exclusion scores between high- and low-risk groups, including density and boxplot visualization. **(E)** Immunophenoscore (IPS) comparison across immune checkpoint subgroups (e.g., CTLA4+/PD1+) in high- and low-risk groups. **(F)** Drug sensitivity predictions derived from the oncoPredict package, showing estimated response to various anticancer drugs across risk groups *P < 0.05; **P < 0.01; ***P < 0.001.

To explore the predictive potential of DCRS in immunotherapy and drug response, we first analyzed the expression of immune checkpoint genes across risk groups. Most inhibitory molecules—including PDCD1, CTLA4, TIGIT, and HAVCR2—were significantly upregulated in the high-DCRS group (**Figure 9A**), indicating enhanced immunosuppressive signaling. Expression correlation analysis showed stronger co-expression patterns among checkpoint genes in the high-DCRS group (**Figure 9B**), suggesting a more unified immunoregulatory profile. Additionally, the high-DCRS group exhibited higher TIDE scores (**Figure 9C**), reflecting a greater potential for immune evasion, as well as elevated exclusion scores (**Figure 9D**), indicative of increased T cell exclusion within the tumor microenvironment. The immunophenoscore (IPS) was used to estimate potential responsiveness to checkpoint blockade therapy. Across multiple immunotherapy scenarios (anti-PD1, anti-CTLA4, or both), the low-DCRS group consistently showed higher IPS values (**Figure 9E**), supporting its association with greater immunogenicity and potential responsiveness.

Drug sensitivity analysis using the oncoPredict framework revealed that patients in the low-DCRS group exhibited significantly lower predicted IC50 values for various chemotherapy and targeted agents (e.g., cisplatin, docetaxel, gemcitabine, erlotinib) (**Figure 9F**). These findings suggest enhanced drug sensitivity in the low-DCRS subgroup, with potential implications for precision therapy selection.

### 3.11 *PLEK2* identified as a functional risk gene and validated as a promoter of LUAD progression

Univariate Cox regression analysis of the DCRS component genes revealed that LDHA and *PLEK2* were the most significant risk factors associated with poor prognosis. Given that the role of LDHA in LUAD has been extensively studied, we selected *PLEK2* for further validation and functional characterization.

**Figure 10:**
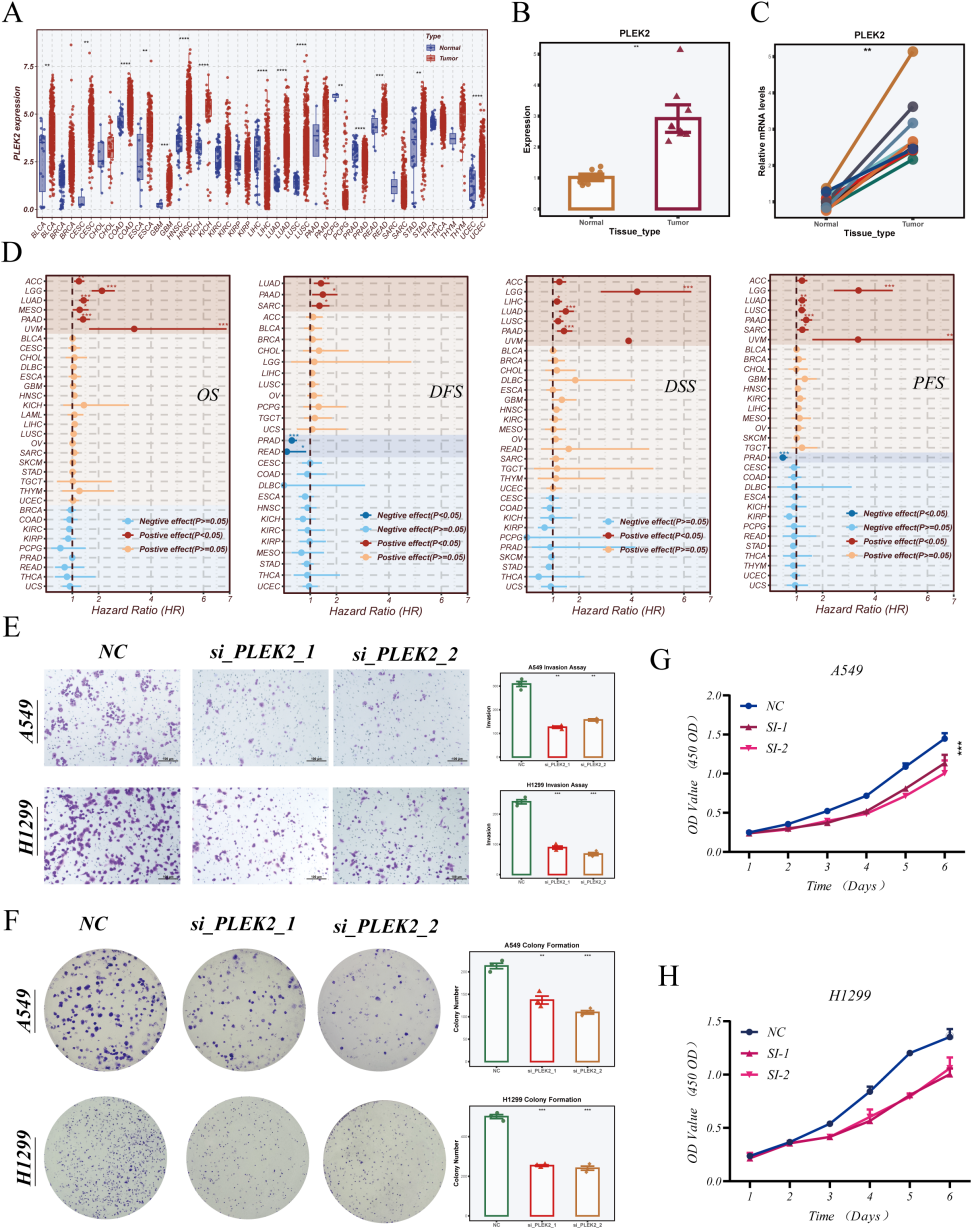
*PLEK2* expression patterns and functional validation in LUAD. **(A)** Pan-cancer analysis of *PLEK2* expression across multiple tumor types based on TCGA datasets. **(B, C)** Relative expression of *PLEK2* in paired tumor and adjacent normal tissues from surgical specimens of LUAD patients collected at Tianjin Chest Hospital, assessed by quantitative PCR. **(D)** Univariate Cox regression analysis of *PLEK2* expression and its prognostic impact (overall survival [OS], disease-free survival [DFS], disease-specific survival [DSS], and progression-free survival [PFS]) across various cancers. **(E)** Transwell invasion assay evaluating the invasive capacity of A549 and H1299 cells following *PLEK2* knockdown using two independent siRNAs. Representative images and quantification are shown. **(F)** Colony formation assay assessing the long-term proliferative ability of A549 and H1299 cells upon *PLEK2* silencing. CCK-8 assay showing time-dependent cell viability in A549 **(G)** and H1299 **(H)** cells after *PLEK2* knockdown. p < 0.05; *p < 0.01; **p < 0.001;***P < 0.001; **** P < 0.0001.

To examine its expression landscape, we performed a pan-cancer analysis using TCGA datasets. As shown in **Figure 10A**, *PLEK2* was significantly upregulated in multiple tumor types, including LUAD. To confirm this observation in clinical specimens, we assessed *PLEK2* expression in paired tumor and adjacent normal tissues from LUAD patients who underwent surgical resection at Tianjin Chest Hospital. qRT-PCR analysis confirmed that *PLEK2* was significantly overexpressed in tumor tissues (**Figures 10B, C**). We then evaluated the prognostic significance of *PLEK2* across human cancers using univariate Cox regression. As shown in **Figure 10D**, high *PLEK2* expression was associated with worse overall survival (OS), disease-free survival (DFS), disease-specific survival (DSS), and progression-free survival (PFS) in several tumor types, including LUAD.

To determine the functional role of *PLEK2* in LUAD, we conducted a series of *in vitro* assays. Transwell invasion assays showed that *PLEK2* knockdown via two independent siRNAs significantly reduced the invasive capacity of A549 and H1299 cells (**Figure 10E**). Colony formation assays demonstrated that *PLEK2* silencing suppressed long-term proliferative ability in both cell lines (**Figure 10F**). Consistently, CCK-8 assays revealed that *PLEK2* knockdown inhibited cell viability in a time-dependent manner (**Figures 10G, H**). Collectively, these findings indicate that *PLEK2* not only correlates with poor clinical outcomes but also plays a functional role in promoting LUAD cell proliferation and invasiveness.

## 4 Discussion

LUAD remains a major contributor to global cancer mortality. Although targeted therapies and ICIs have significantly improved patient outcomes, durable clinical benefit is only achieved in a limited subset of patients. This disparity is largely attributed to the complex immunosuppressive TME, profound intertumoral heterogeneity, and immune evasion mechanisms that remain poorly understood (8, 9). One of the underexplored yet critical components of the LUAD immune landscape is the DC compartment (33).

DCs play a pivotal role in initiating and shaping adaptive immune responses by capturing, processing, and presenting antigens to T cells (34). However, emerging studies have highlighted the extensive heterogeneity within DC populations, including conventional DC1 (cDC1), cDC2, plasmacytoid DCs (pDCs), and monocyte-derived DCs (MoDCs) (35). Each subtype exhibits distinct ontogeny, transcriptional programs, and immunological functions. In LUAD, cDC1 are often depleted or functionally impaired, compromising CD8+ T cell priming (36); cDC2 may either support Th responses or acquire suppressive properties (37); pDCs secrete type I interferons but may paradoxically promote immune evasion; and MoDCs often contribute to chronic inflammation and immunosuppression. Dysfunctional differentiation, reduced migration, and impaired maturation of DCs collectively contribute to immune escape and resistance to immunotherapy (35).

Compared with traditional transcriptomic approaches, scRNA-seq provides an unprecedented opportunity to dissect the cellular and functional diversity of tumor-infiltrating DCs (38). Unlike previous studies that primarily relied on bulk transcriptomic data, our approach leverages single-cell resolution to unravel the phenotypic and functional heterogeneity of DCs in LUAD with higher precision. It enables fine-grained delineation of rare subsets, developmental trajectories, and functional states at the single-cell level (39). In particular, integrating scRNA-seq with pseudotime inference and hdWGCNA allows for the systematic mapping of transcriptional modules associated with DC heterogeneity and immunological dysfunction. This framework not only advances our understanding of DC biology but also facilitates the development of clinically relevant prognostic models grounded in biological context. In this study, we established a comprehensive DC landscape in LUAD using scRNA-seq and identified two transcriptionally and functionally distinct DC clusters. One cluster was enriched in immune-activating pathways (e.g., IFN-α, TNF/NF-κB), while the other exhibited signatures associated with proliferation and cell cycle. These clusters demonstrated significant prognostic value, reflecting the dual roles of DCs in immune activation and immunosuppression.

Based on these findings, we constructed a DCRS by integrating marker genes from key DC clusters and co-expression modules with machine learning algorithms. The DCRS model demonstrated robust prognostic performance across seven independent validation cohorts, consistently outperforming traditional clinical features and previously published LUAD signatures. This advantage is largely attributed to the biologically informed model design, which integrates DC-specific markers derived from co-expression networks rather than relying solely on statistical associations. In addition to predicting survival, DCRS stratified immune infiltration patterns, chromosomal instability, and therapeutic response profiles. These results highlight DCRS as a biologically informed and technically rigorous tool for immune-based stratification in LUAD.

Among the genes incorporated in DCRS, *PLEK2* was identified as a hub gene with both prognostic and functional significance. *PLEK2* encodes pleckstrin-2, a protein involved in cytoskeletal remodeling and cellular motility (40). While *PLEK2* has been implicated in EMT and metastasis in several cancers, its role in LUAD has been less explored (41–43). In our study, *PLEK2* was significantly overexpressed in tumor tissues and promoted proliferation, migration, and colony formation in LUAD cell lines. These findings suggest that *PLEK2* may function as an oncogenic effector in LUAD. Furthermore, given its role in actin dynamics, *PLEK2* may influence DC mobility and antigen-presenting capacity, warranting further investigation into its immunological functions.

Despite these strengths, several limitations must be acknowledged. First, our model was constructed and validated using retrospective datasets; prospective, multicenter validation is necessary to confirm its clinical utility. Second, although we demonstrated the oncogenic role of *PLEK2* in LUAD cells, its precise impact on dendritic cell biology and immune regulation remains undefined. Third, the spatial organization of DC subsets within the TME, such as their distribution in tertiary lymphoid structures or invasive margins, was not addressed due to the lack of spatial transcriptomics data.

Future studies should aim to integrate spatially resolved technologies to explore the localization and intercellular interactions of DCs in LUAD. Functional dissection of *PLEK2* in specific DC subsets, using *in vitro* co-culture systems and *in vivo* models, may also uncover novel regulatory pathways involved in immune evasion. Additionally, DCRS holds promise not only as a prognostic tool but also as a potential predictor of immunotherapy response and a guide for DC-targeted therapeutic strategies.

In conclusion, our study proposes a novel dendritic cell–related signature that captures the complexity of DC heterogeneity and provides mechanistic insights into LUAD progression and immune modulation. The identification of *PLEK2* as a functional driver further strengthens the translational relevance of the model. These findings contribute to the growing interest in myeloid-targeted immunotherapy and offer a roadmap for DC-centered personalized medicine in LUAD.

## Data Availability

The original contributions presented in the study are included in the article/**Supplementary Material**. Further inquiries can be directed to the corresponding author.

## Supplementary material

Supplementary Figure 1 Quality control and preprocessing of single-cell RNA-sequencing data. **(A)** Violin plots showing the distribution of RNA features (nFeature_RNA), total UMI counts (nCount_RNA), and mitochondrial gene percentage (pMT) across all cells before quality control. **(B)** Violin plots of the same metrics after quality control filtering. Cells with extreme feature counts or high mitochondrial content were removed. **(C)** Principal component analysis (PCA) plot before cell cycle correction, colored by cell cycle phase (G1, S, and G2M). **(D)** PCA plot after regressing out cell cycle effects using the ScaleData function in Seurat, showing reduced separation driven by cell cycle-related variance. Distribution of cells from different samples before **(E)** and after **(F)** batch effect correction using the Harmony algorithm, visualized by UMAP. Cells from distinct samples exhibit better integration following batch correction.

Supplementary Figure 2 Seven co-expression modules (M1–M7) were identified using the hdWGCNA framework and are depicted in different colors. Each node represents an individual gene, and edges denote co-expression relationships between genes.

Supplementary Figure 3 Comprehensive Evaluation of Risk Stratification, Molecular Interactions, and Clinical Stage Patterns. **(A)** Risk score distribution and overall survival status of patients based on the DCRS signature. The upper panel displays the distribution of individual risk scores, stratifying patients into high- and low-risk groups. The middle panel shows the corresponding survival time and status. The bottom heatmap visualizes the expression profiles of genes comprising the DCRS signature across patients. **(B)** Correlation matrix depicting the associations between risk scores and individual gene expressions within the DCRS signature. Positive and negative correlations are indicated by the color gradient. **(C)** Distribution of pathological stages between high- and low-risk groups.
